# A Study on the Reference Values and Cutoff Criteria of Masking Level Difference for Children Aged 7–12 Years

**DOI:** 10.3390/jcm11185282

**Published:** 2022-09-07

**Authors:** Carlos Alberto Leite Filho, Mônica de Oliveira Viana, Fátima Cristina Alves Branco-Barreiro, Silvana Maria Monte Coelho Frota

**Affiliations:** 1 Department of Physical Therapy, Speech-Language Pathology and Occupational Therapy, School of Medicine, University of São Paulo, São Paulo 05360-160, Brazil; 2 Department of Speech Therapy, Medicine Faculty, Federal University of Rio de Janeiro, Rio de Janeiro 21941-617, Brazil; 3 Department of Speech Therapy, Paulista School of Medicine, Federal University of São Paulo, São Paulo 04023-062, Brazil

**Keywords:** masking level difference, binaural interaction, central auditory processing, central auditory processing disorder, hearing tests, auditory perception

## Abstract

The Masking Level Difference (MLD) test is one of the main instruments for investigating binaural interaction. Studies with children aged 7–12 years still disagree about the influence of age on test performance and present discordant reference values. This study aimed to verify the effect of age on the performance of children aged 7–12 years in the MLD test and to establish reference values and cutoff criteria for this age group. Fifty-nine children with normal hearing were organized in three groups according to their age: 7–8 (n = 20), 9–10 (n = 20), and 11–12 (n = 19) years. The participants completed the MLD test by Auditec^®^. The Kruskal–Wallis statistical test was used to compare groups. Reference values were obtained by calculating mean, standard deviation, median, mode, and percentiles, while the cutoff criterion was obtained by subtracting two standard deviations from the mean. No statistically significant differences were observed between the groups regarding the MLD test measures. The mean MLD was 10.51 ± 1.84 dB and the cutoff point was set at 7 dB. Thus, reference values for the MLD test were established for children aged 7–12 years, who presented no effect of age on test performance.

## 1. Introduction

According to the American Speech-Language-Hearing-Association (ASHA) [1], central auditory processing (CAP) can be defined as the perceptual processing of auditory information and includes the neurobiological activity of the central auditory nervous system (CANS). Individuals with central auditory processing disorder (CAPD) may present characteristics such as difficulty paying attention, being easily distracted, longer response times, language and reading impairments, and difficulty performing auditory tasks, such as locating sound, hearing in noise, understanding rapid speech, and following verbal commands, among others. Due to the close relationship between CAP and the development of language and academic abilities, as well as the positive effect of CAPD interventions on academic and language performance, the population of school-aged children is the main focus of clinical practice on CAP and its assessment is recommended for children aged seven years or older [2,3,4].

Due to the multidimensional quality of CAP, its assessment consists of a battery of behavioral tests aiming to evaluate different auditory abilities. Binaural interaction is an auditory ability characterized by the processing of sounds, which may or may not be complementary, presented binaurally. By allowing the fusion of such stimuli in a single acoustic event, this skill facilitates tasks such as sound localization, hearing in noise, and hearing with competitive stimuli [5,6]. One of the main markers of the integrity of binaural interaction is the presence of a phenomenon known as release from masking, which was firstly described by two simultaneous studies conducted with tonal [7] and verbal [8] stimuli accompanied by noise. This phenomenon is characterized by the improvement of the auditory threshold for a tonal or verbal stimulus through its presentation in an antiphasic condition on one ear (i.e., phase-reversed in relation to the same stimulus on the other ear) in comparison to a homophasic condition (i.e., same phase on both ears) [9,10]. Studies with electrophysiological procedures and individuals with neurological lesions suggest that the brainstem is the main CANS structure related to binaural interaction [9,11,12].

The main CAP guidelines argue that the scientific community must turn its efforts towards developing more reliable, valid, and efficient tests to be used for the diagnosis of CAPD [1,2,13,14,15]. One of the main paradigms for assessing release from masking is Masking Level Difference (MLD), which received a clinically feasible version in 2003 [16]. The MLD presents good sensitivity and specificity for CAPD [17] and a recent study demonstrated satisfactory test-retest reliability for clinical use [18]. Additionally, MLD suffers from little or no influence of aspects such as parental schooling, socioeconomic status [19], memory, attention, cognition [20], or language [21]. Such features, along with the use of non-verbal stimuli and easy utilization, make the MLD one of the main clinical tools for assessing CAP.

However, some properties of the MLD are still lacking consensus among researchers and need to be further explored. One of these properties is the change in performance seen throughout the development of hearing in children aged 7–12 years. While some studies point out that performance on the MLD remains stable throughout this age range [19,21,22,23,24,25,26], possibly due to the early (i.e., pre-school years) maturation of binaural interaction and the brainstem [27], other works observed a relationship between increasing age and performance improvement in this age group [28,29,30], reflecting a more recent study suggesting that the auditory function at the brainstem changes across the lifespan [31].

Another issue of the MLD refers to the reference values and normality criteria for children aged 7–12 years. Different studies found significantly discrepant magnitudes for the phenomenon of release from masking which is observed in the MLD, with a hearing threshold improvement in the antiphasic condition ranging from 6.95 to 14.60 dB in comparison to the homophasic condition [19,22,23,24,26,30,32]. Regarding normality criteria, investigations using the “mean minus 2 standard deviations (SD)” method recommended by most CAPD guidelines [1,2,13,14,15] suggested the cutoff points of 7.87 [26] and 9.30 dB [22]. However, depending on factors such as the psychometric properties of each test, the patient’s clinical history, and performance in other CAP tests, diverse cutoff criteria have been suggested, such as percentile 2.5 [32], percentile 10 [33,34], mean minus 1 or 1.5 SD [35], and mean minus 3 SDs [1,2].

To guarantee the validity of the MLD as a CAPD diagnosis tool, as recommended by the scientific guidelines of the field, such problems must be addressed. In addition, a better understanding of children’s performance on the MLD may help to elucidate the maturation of binaural interaction, as well as ensure early diagnosis and the effective intervention in patients with CAPD in order to mitigate the detrimental effects imposed by this clinical condition. Thus, this study aims to investigate the effect of age on the MLD performance in children aged 7–12 years and to establish reference values and cutoff points for this age group.

## 2. Materials and Methods

This is a prospective, descriptive study conducted at the Clementino Fraga Filho University Hospital of the Federal University of Rio de Janeiro. The convenience sample was composed of 59 children, 20 of which were 7 and 8 years old (eight girls, mean age = 7.55 ± 0.51 years), while 20 were 9 and 10 years old (eleven girls, mean age = 9.25 ± 0.44 years), and 19 were 11 and 12 years old (six girls, mean age = 11.74 ± 0.45 years). These children were users of the Unified Health System and were recruited to participate in this research after the verification of normal hearing thresholds (≤20 dBHL, 0.25–8 kHz and absence of air-bone gap) and absence of the following: auditory, language, or reading complaints, recent otological impairments or surgeries, acoustic trauma, and neurological disorders. After the inclusion phase of the study, each child completed a CAP screening which included the Brazilian Portuguese version of the Dichotic Digit Test (DDT) [36,37,38]. Only children with performance strictly within normal limits for the DDT, as proposed by Pereira and Schochat [39], were included in this investigation.

The children who fit the inclusion and exclusion criteria described above were submitted to the Auditec ® version of the MLD (Auditec, Inc., St. Louis, MO, USA). This version is characterized by the presentation of 33 acoustic stimuli of narrowband noise in both ears, with a minimum duration of three seconds, which may or may not be accompanied by a 500 Hz pulsatile pure tone, in various signal-to-noise ratios (SNR). The test items are presented in three different conditions: pure tone and noise on the same phase in both ears (homophasic condition—S_0_N_0_); phase-reversed pure tone in one ear and noise on the same phase in both ears (antiphasic condition—S_π_N_0_); and noise without pure tone (no tone—NT). Immediately after each item, the children should indicate whether they heard the tone by raising their hand. The test was performed binaurally with an intensity level of 50 dBHL [40].

For each child, test performance was analyzed by quantifying the hits for each condition and converting this number to its corresponding SNR in each condition’s hearing threshold by using the conversion table available in the test manual. The final result (i.e., the MLD) was calculated by subtracting the SNR in the hearing threshold for S_π_N_0_ condition (SNR-S_π_N_0_) from the SNR in the hearing threshold for S_0_N_0_ condition (SNR-S_0_N_0_) [40].

Equipment used in this study included Madsen Itera II audiometer (GN Otometrics, Schaumburg, IL, USA), a compact disc containing the recordings for the DDT and the MLD test, and a CD player (Samsung do Brasil, Manaus, AM, Brazil) coupled to the audiometer.

SPSS Statistics software (IBM Corp., Armonk, NY, USA), version 25.0, was used to perform statistical analysis, which was conducted according to the guidelines determined by Field [41]. Due to the violation of the assumption of normality of data distribution in at least one of the groups (*p* ≤ 0.05, Shapiro–Wilk test), the age groups were compared through non-parametric procedures, namely, the Kruskal–Wallis H test. The relationship between age and MLD performance was also investigated by running correlation analyses between age and MLD parameters through the Pearson’s correlation test with the Bonferroni correction for multiple correlations [42]. Then, descriptive analysis was conducted by calculation of central tendency and dispersion measures, and data distribution was analyzed by plotting histogram charts and Q–Q plots for each MLD parameter. The Q–Q plots complemented the information provided by the histogram charts by comparing the observed variables distribution to a hypothetical normal distribution, so that values falling on the diagonal lines demonstrated correspondence between observed and hypothetical distributions. Cutoff points were calculated by using means and SDs for each MLD parameter, as preconized by the main guidelines of the field [1,2]. Bias-corrected and accelerated 95% confidence intervals (BCa 95% CI) are reported in brackets in the tables and were based on 2000 bootstrap samples.

## 3. Results

**Figure 1:**
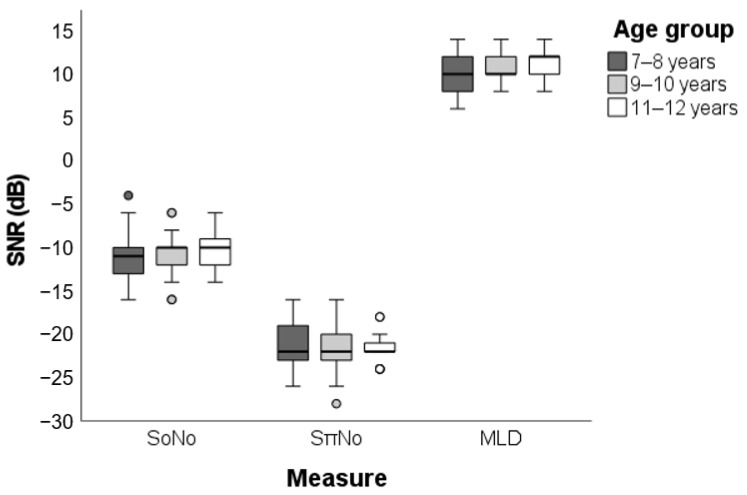
Children’s performance by age group in the S_0_N_0_ and S_π_N_0_ conditions and in the final MLD. SNR: signal-to-noise ratio; S_0_N_0_: homophasic condition; S_π_N_0_: antiphasic condition; MLD: Masking Level Difference.

No statistically significant differences were found between the three age groups regarding the SNR-S_0_N_0_ (H (2) = 0.778, *p* = 0.678) and SNR-S_π_N_0_ (H (2) = 0.208, *p* = 0.901), as well as for the final MLD value (H (2) = 3.300, *p* = 0.192) (Figure 1). Besides that, no statistically significant correlation was observed between age and SNR-S_0_N_0_ (r = 0.191, BCa 95% CI = [−0.057, 0.425], *p* = 0.438), SNR-S_π_N_0_ (r = 0.003, BCa 95% CI = [−0.263, 0.257], *p* > 0.999), and MLD (r = 0.272, BCa 95% CI = [−0.014, 0.520], *p* = 0.111). Thus, the participants were reunited in a single group containing 59 individuals in order to increase the statistical power of the sample.

The SNR-S_0_N_0_ ranged from −16 to −4 dB, while the SNR-S_π_N_0_ ranged from −28 to −14 dB and the final MLD ranged from 6 to 14 dB. As for the values within the central 95% of the data, these were between −16 and −5 dB for the SNR-S_0_N_0_, −27 and −15 dB for the SNR-S_π_N_0_, and 7 and 14 dB for the MLD (Table 1).

**Figure 2:**
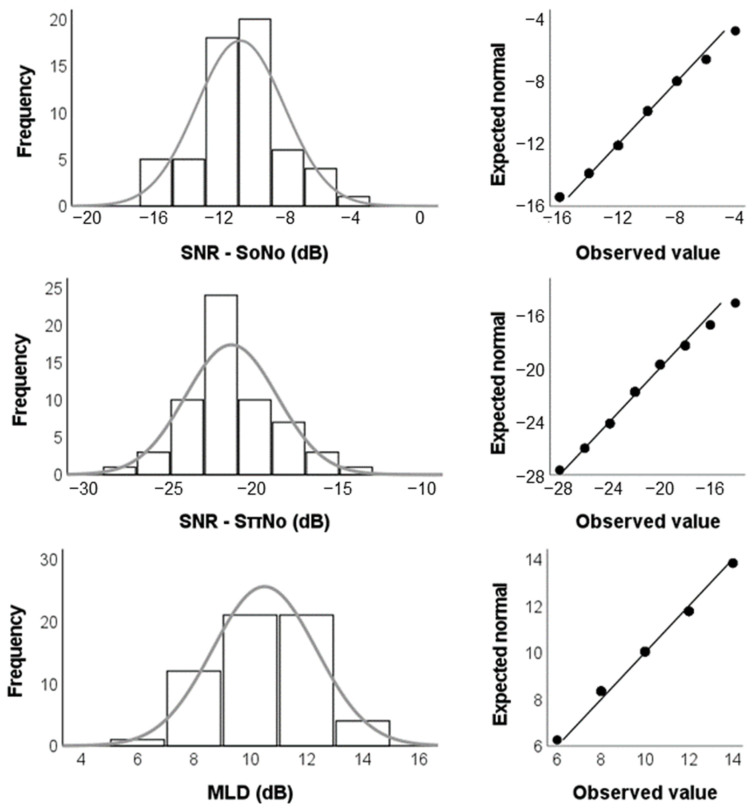
Histogram charts and Q–Q plots of the study data for each MLD parameter. SNR: signal-to-noise ratio; S_0_N_0_: homophasic condition; S_π_N_0_: antiphasic condition; MLD: Masking Level Difference.

Histogram charts, as well as Q–Q plots, regarding the MLD parameters for the total sample revealed a data distribution similar to the normal curve (Figure 2). Therefore, cutoff points based on the mean and SD values were calculated. For the final MLD value, the cutoff point based on the “mean minus 2 SDs” method was 7 dB (Table 2).

## 4. Discussion

We found no statistically significant differences between the three different age groups regarding performance at the MLD test (Figure 1), as well as no significant correlations between age and MLD performance. The final MLD mean was 10.51 dB, with a standard deviation of 1.84 (Table 2) and the central 95% of the data ranging between 7 and 14 dB (Table 1).

The similarity of the mean MLD for the different age groups of the present study corroborates the results of other investigations which observed no correlation between age and the MLD in children aged 5–12 years [19,21,22,23,24,25,26]. However, such results disagree with other works which reported an association between age and performance in the MLD test [28,29,30]. A possible explanation for such disparity may reside in the fact that the MLD paradigm used in the latter studies differs from the Auditec^®^ version in terms of stimulus type, item number, response type, etc. In addition, one of these studies [30] included children aged three to four years, possibly introducing a maturational bias which might not be present in other studies investigating children aged five or older.

The MLD stability across different age groups can be explained by the early maturation of binaural interaction, as demonstrated by studies which observed adult-like MLD mean values in pre-school children [43,44]. The MLD is closely related to the brainstem function [9,11,12], one of the first CANS structures to reach its full maturation, which may explain the early maturation of binaural interaction in comparison to other auditory skills [27]. In accordance with this hypothesis, the mean MLD observed in the present study was similar to another investigation with Brazilian adults (10.83 dB) [45], as well as the mean minus 2 SDs cutoff point for adults (7 dB) [46].

By comparing the 95% confidence interval for the present work with other investigations, we verified that the mean MLD for this study is similar to that of an Australian publication with children with similar age [26]. However, other studies showed significant discrepancies. Two Brazilian studies [19,24] reported lower MLD means, while four studies conducted in different countries [22,23,30,32] reported higher values. Additionally, due to SD differences between these works, even similar MLD means resulted in divergent cutoff points, which may ultimately lead to disagreements between clinicians with different reference values (Table 3).

Thus, previous research intending to set reference values for the MLD test present high heterogeneity, which may be justified by factors such as small sample size and uncontrolled biases which may impact on the participants’ performance. Even though there are evidences suggesting that the MLD is not influenced by variables such as gender [46], family income, parental schooling [19], language experience [21], and cognitive abilities [20]; other aspects, such as musical training [48] and sleep-disordered breathing [49,50], may impact auditory abilities and, so far, have not been properly controlled in research investigating reference values for the MLD test. Therefore, new investigations with a more rigorous control of these and other variables must be done in order to reduce the variability of results and obtain reference values with more reliability.

Many guidelines for clinical practice in CAP present the mean minus two SDs cutoff point as appropriate for the identification of individuals with CAPD [1,2,13,14,15] due to the higher balance between sensitivity and specificity provided by this method [51,52]. In the present study, the corresponding mean minus 2 SDs cutoff point for the final MLD was 7 dB for children aged 7–12 years.

Although this is the most accepted cutoff point calculation method, it should be noted that there are other proposals in the literature which may be more suitable in certain situations in clinical practice [51]. Some guidelines suggest that, in cases with poor performance observed on only one test of the CAP assessment battery, CAPD diagnosis must be considered only if the performance falls at least three SDs below the mean [1,2,15]. In this context, considering the data obtained by the present research, individuals with an MLD below 5 dB would be considered abnormal. On the other hand, cutoff points based on percentile 2.5 [32] and percentile 10 [33,34] would be set at 7 and 8 dB, respectively. Finally, a more recent proposal [35] suggests that the cutoff points must be set after 1 or 1.5 SD below the mean, which is equivalent, in the present work, to 9 and 8 dB, respectively.

The choice of the diagnostic criterion for CAPD depends on many factors, including patient’s medical history, weighting of sensitivity and specificity during the interpretation of results, the battery of tests used in the assessment, and the data available for establishment of cutoff points [1,2,15,51]. However, regardless of the chosen criterion, to make this choice clear is fundamental in the clinical practice, since it may lead to diagnostic variability [53].

Among the limitations of the present study, the lack of control for possible confounding variables, such as gender, socioeconomic status, second language study, and cognitive abilities, can be cited. However, as previously stated, literature points out that these variables are not related to performance in the MLD test. Another limitation refers to the absence of a study group, composed of individuals with CANS lesion or CAPD, to empirically test the cutoff points. Nonetheless, we tried to overcome this limitation by employing a well-accepted cutoff point (mean minus 2 SDs) for detecting individuals with CAPD with similar efficiency to cutoff points obtained by other methods [54]. Finally, the sample size per age group was reduced and future studies with bigger samples and other age groups (e.g., children aged six years or younger) may provide deeper insights towards the age effect on the MLD test, contributing to early diagnosis and intervention on CAPD.

Thereby, we believe that this study presents a relevant contribution to the recent efforts put forward by many research groups globally to adequately establish reference values for tests used in the behavioral CAP assessment and, thus, promote better validity and accuracy for the CAPD diagnosis.

## 5. Conclusions

Children with ages between 7 and 12 years had similar results in the MLD test. The mean value obtained was 10.51 ± 1.84 dB. Considering the mean minus two SDs method, a cutoff point of 7 dB is suggested for this age group.

## Figures and Tables

**Table 1:** Descriptive analysis of S_0_N_0_ e S_π_N_0_ conditions and of the final MLD regarding mode and percentiles.

Parameters	Mode	Percentile								
		2.5	5	10	25	50	75	90	95	97.5
SNR-S_0_N_0_ (dB)	−10	−16	−16	−14	−12	−10	−10	−8	−6	−5
SNR-S_π_N_0_ (dB)	−22	−27	−26	−24	−22	−22	−20	−18	−16	−15
MLD (dB)	10	7	8	8	10	10	12	12	14	14

SNR: signal-to-noise ratio; S_0_N_0_: homophasic condition; S_π_N_0_: antiphasic condition; MLD: Masking Level Difference.

**Table 2:** Descriptive analysis of the MLD parameters regarding means, standard deviations, and cutoff points.

Measure	Mean [BCa 95% CI]	SD			Cutoff Point (SD)					
			−3	−2	−1.5	−1	+1	+1.5	+2	+3
SNR-S_0_N_0_ (dB)	−10.88 [−11.49, −10.26]	2.66	--	--	--	--	−8.22	−6.89	−5.56	−2.90
SNR-S_π_N_0_ (dB)	−21.39 [−22.03, −20.75]	2.71	--	--	--	--	−18.68	−17.32	−15.97	−13.26
MLD (dB)	10.51 [10.04, 10.95]	1.84	4.99	6.83	7.75	8.67	--	--	--	--

SNR: signal-to-noise ratio; S_0_N_0_: homophasic condition; S_π_N_0_: antiphasic condition; MLD: Masking Level Difference; SD: standard deviation; BCa 95% CI: bias-corrected and accelereated 95% confidence interval.

**Table 3:** Comparison of results from other studies investigating reference values for the MLD in children and estimates of 95% CIs and mean—2 SDs.

Study (Data Source)	Age Group	n	Mean	SD	95% CI ^b^		Mean—2 SD ^b^
					LL	UL	
Leite Filho et al. (Present study)	7–12 years	59	10.51	1.84	10.04	10.95	6.83
Aithal et al. [26]	6–13 years	62	11.21	1.67	10.79	11.63	7.87
Moore et al. [23]	6–11 years	45	13.88 ^a^	4.61 ^a^	12.49	15.27	4.66
Porter et al. [30]	3–12 years	46	13.70	4.90	12.28	15.12	3.90
Gicov et al. [24]	7–8 years	21	6.95	2.33	5.95	7.95	2.29
Martins et al. [19]	7–10 years	31	7.65	2.51	6.77	8.53	2.63
Mattsson et al. [32]	7–12 years	266	14.60	2.80	14.26	14.94	9.00
de Carvalho et al. [22]	7–12 years	47	13.66	2.18	13.04	14.28	9.30

MLD: Masking Level Difference; SD: standard deviation; CI: confidence interval; LL: lower limit; UL: upper limit; ^a^: these values were not available in the original study and were estimated by using the median, minimum, maximum and quartile values as proposed by Wan et al. [47]; ^b^: these values were not available in the original study (except for Aithal et al. [26] and de Carvalho et al. [22]) and were estimated after the mean and standard deviation values.

## Data Availability

The data presented in this study are available on request from the corresponding author. The data are not publicly available due to ethical issues.
